# Infective Endocarditis Presenting As Meningeal Syndrome in an Intravenous Drug User: A Diagnostic Challenge

**DOI:** 10.7759/cureus.90066

**Published:** 2025-08-14

**Authors:** Idalberto Luis Fernandez Eng, Eliany Leon Figueredo, Alicia De Fuenmayor Icardo, Idania Maria Cruzata Matos, Yoniel Suarez-Guerrero

**Affiliations:** 1 Emergency, Hospital Universitario de la Ribera, Valencia, ESP; 2 General Medicine, University of Medical Sciences Cienfuegos, Cienfuegos, CUB; 3 Radiology Resident, Hospital Universitario de la Ribera, Valencia, ESP; 4 General Medicine, Facultad de Ciencias Medicas, Holguin, CUB; 5 General Medicine, Asociacion Española de Socorros Mutuos, Montevideo, URY

**Keywords:** infective endocarditis, intravenous drug use, meningeal syndrome, mitral valve surgery, staphylococcus aureus, subarachnoid hemorrhage

## Abstract

Infective endocarditis (IE) commonly presents with fever, fatigue, and signs of systemic infection. However, atypical neurological manifestations may delay diagnosis, especially in high-risk populations such as intravenous drug users (IVDUs).

We report the case of a 48-year-old man with a history of intravenous drug use who presented with fever, abdominal pain, and altered mental status. On examination, the patient had neck stiffness, a petechial rash, and positive Kernig’s and Brudzinski’s signs, all of which supported the presence of meningeal irritation. Laboratory studies revealed thrombocytopenia, elevated inflammatory markers, and abnormal liver enzymes. Imaging revealed subarachnoid hemorrhage, splenic and renal infarctions. The patient was admitted to intensive care, where he received broad-spectrum antibiotics and supportive therapy. Blood cultures grew *Staphylococcus aureus*. A cerebrospinal fluid analysis showed signs of meningeal inflammation, and a transthoracic echocardiogram identified a mitral valve vegetation. Despite initial treatment with broad-spectrum antibiotics, the patient persisted with fever and eventually required mitral valve replacement.

This case underscores the critical need to maintain a high index of suspicion for left-sided infective endocarditis in IVDUs presenting with septic syndromes and multiorgan involvement. Importantly, the presence of meningeal signs in this patient played a key role in early recognition of central nervous system involvement. IE should be considered even when clinical features initially suggest alternative diagnoses, such as meningeal syndrome, as timely recognition and initiation of appropriate therapy are essential to improve patient outcomes.

## Introduction

IE is a serious infection of the endocardial surface, usually involving heart valves, caused by bacteria or fungi. Vegetations composed of microorganisms, fibrin, and platelets can lead to systemic embolization, affecting organs such as the brain, lungs, and kidneys. Clinical presentation varies from fever and a new murmur to stroke, meningitis, or pneumonia. Despite improvements in diagnostics like blood cultures and echocardiography, early detection remains challenging. Without timely treatment, IE can result in severe complications or be fatal [1].

In the United States, the prevalence of IE has steadily increased over time. While it remained relatively stable between 1970 and 2000, ranging from 30 to 100 cases per 100,000 people, a noticeable rise began after 2000. From 2000 to 2011, the annual incidence climbed from 11 to 15 cases per 100,000, despite updates to prophylactic guidelines. More recent data from the 2019 report an incidence of approximately 13.8 per 100,000 individuals, reflecting a persistent upward trend driven by changes in healthcare practices, population aging, and intravenous drug use (IVDU). This increase is not uniform across all populations; subgroups such as elderly patients, those with prosthetic valves, and IVDUs have shown particularly marked rises in incidence [2].

IVDU is acknowledged as a risk factor for developing IE and is classified as a minor criterion in the Duke diagnostic framework. Although the implementation of harm reduction measures and a shift toward non-injectable drug use had previously contributed to a decline in IE incidence among this population, recent data from the United States indicate an increasing number of hospitalizations for IE, with some reports highlighting a disproportionate rise among young white women, although this demographic trend remains under investigation [3].

Right-sided endocarditis is most common among IVDU, typically caused by *Staphylococcus aureus*. This right-sided involvement is often related to direct invasion of the venous bloodstream and predominantly affects the tricuspid and pulmonary valves. In contrast, left-sided involvement is associated with more severe outcomes, including higher rates of systemic embolization, heart failure, need for surgical intervention, and increased mortality. Studies show that IVDU patients with left-sided disease are often younger, have multivalve involvement, recurrent infections, prior endocarditis, and a higher risk of stroke [4]. Additionally, other causative agents in the general population include *Haemophilus spp., Aggregatibacter actinomycetemcomitans, Cardiobacterium hominis, Eikenella corrodens, *and *Kingella kingae* (HACEK) organisms, which are gram-negative bacteria part of the normal oral and upper respiratory flora and recognized pathogens in infective endocarditis [1].

Systemic embolization is a common complication of IE, with the brain and spleen being frequent targets in left-sided IE. Embolic events to the central nervous system occur in approximately 25% to 40% of cases and are linked to poor outcomes. Among these, meningitis is a severe manifestation characterized by inflammation of the meninges, which clinically presents with signs of meningeal irritation such as nuchal rigidity, Kernig’s sign, and Brudzinski’s sign [5,6]. Brain abscesses occur more frequently in acute than subacute endocarditis and can present as space-occupying lesions, toxic encephalopathy, or meningitis. Splenic, renal, and some cerebral embolisms are often asymptomatic and detected through routine diagnostic tests when searching for distant complications [7].

Atypical presentations, particularly neurological symptoms, can obscure the diagnosis and delay treatment. This case underscores the importance of considering IE in IVDU presenting with non-specific or neurological complaints, even when the clinical picture includes meningeal syndrome.

## Case presentation

A 48-year-old man with a known history of intravenous drug use and no prior cardiac or known valvular disease presented to the emergency department with a one-week history of fever and abdominal pain. On presentation, his vital signs were notable for fever (39.2°C), tachycardia (115 bpm), hypotension (90/60 mmHg), tachypnea (24 breaths per minute), and oxygen saturation of 96% on room air. On physical examination, the patient appeared disoriented and exhibited signs of meningeal irritation, including nuchal rigidity, photophobia, as well as positive Kernig’s and Brudzinski’s signs, along with petechial lesions on the lower extremities. Cardiac examination was unremarkable, and dermatologic examination revealed petechial lesions on both lower extremities.

Initial laboratory investigations, obtained upon admission to the intensive care unit (ICU), revealed thrombocytopenia, markedly elevated inflammatory markers, and mildly increased liver enzymes. Gamma-glutamyl transferase and bilirubin levels were also elevated, and urinalysis showed hematuria with marked pyuria (Tables 1, 2). Urine toxicology tested positive for cocaine. 

**Table 1:** Laboratory values on admission to the emergency department. GOT: Glutamic oxaloacetic transaminase (AST, aspartate aminotransferase), GPT: Glutamic pyruvic transaminase (ALT, alanine aminotransferase), GGT: Gamma-glutamyl transferase, MCV: Mean Corpuscular Volume, MCH: Mean Corpuscular Hemoglobin, MCHC: Mean Corpuscular Hemoglobin Concentration, RDW: RBC Distribution Width, MPV: Mean Platelet Volume, INR: International Normalized Ratio, fL: Femtoliters, pg: Picograms.

Test	Result	Reference Range
Glucose	101 mg/dL	74 - 106 mg/dL
Creatinine	0.97 mg/dL	0.7 - 1.3 mg/dL
Urea	48 mg/dL	19 - 49 mg/dL
Total Cholesterol	105 mg/dL	< 200 mg/dL
Triglycerides	226 mg/dL	< 150 mg/dL
GOT (AST)	48 U/L	< 34 U/L
GPT (ALT)	90 U/L	< 40 U/L
Alkaline Phosphatase	96 U/L	46 - 116 U/L
GGT	168 U/L	0 - 73 U/L
Total Bilirubin	1.77 mg/dL	0.3 - 1.2 mg/dL
Direct Bilirubin	1.15 mg/dL	0 - 0.3 mg/dL
Indirect Bilirubin	Not calculable	0.2 - 1.0 mg/dL
Total Proteins	4.7 g/dL	5.7 - 8.7 g/dL
Albumin	2.3 g/dL	3.2 - 4.8 g/dL
Sodium	142 mmol/L	136 - 145 mmol/L
Potassium	3.8 mmol/L	3.6 - 5.1 mmol/L
Magnesium	1.7 mg/dL	1.6 - 2.6 mg/dL
Phosphate	3.4 mg/dL	2.4 - 5.1 mg/dL
Calcium	7.8 mg/dL	8.7 - 10.4 mg/dL
Chloride	110 mmol/L	99 - 110 mmol/L
C Reactive Protein	297.9 mg/L	0 - 5.0 mg/L
Procalcitonin	6.49 ng/mL	< 0.5 ng/mL
Red Blood Cells (RBC)	3.36 x 10^6/µL	4.5 - 5.9 x 10^6/µL
Hemoglobin	10.4 g/dL	12.5 - 17.5 g/dL
Hematocrit	30%	40 - 54 %
MCV	89.3 fL	80 - 99 fL
MCH	29 pg	27 - 34 pg
MCHC	30.9 g/dL	26 - 34 g/dL
RDW	14.7%	11.5 - 15 %
White Blood Cells	10.2 x 10^9/L	4.5 - 11.0 x 10^9/L
Neutrophils	80.2 %	40 - 76 %
Lymphocytes	9.7 %	20 - 52 %
Monocytes	9.1%	2 - 12 %
Eosinophils	0.8 %	0 - 7 %
Basophils	0.2 %	0 - 3 %
Platelets	58 x10^9/L	120 - 450 x 10^9/L
MPV	9.6 fL	7 - 11.2 fL
Prothrombin Time	13.5 seconds	9.6 - 14.4 seconds
Quick Time	82 %	70 - 120 %
INR (International Normalized Ratio)	1.1 r	0.9 - 1.2 ratio
Fibrinogen	882 mg/dL	276 - 471 mg/dL

**Table 2:** Urine sediment analysis showing intense pyuria and microscopic hematuria, with trace proteinuria. Parameters are expressed per high-power field (HPF).

Parameter	Result	Reference Range
Appearance	Turbid	Clear
Color	Yellow	-
Specific gravity	1.020	1.005 – 1.030
pH	6	4.5 – 8.0
Leukocytes (WBC)	Intense pyuria	0 – 5 /HPF
Erythrocytes (RBC)	20 - 25 /HPF	0 – 2 /HPF
Protein	Trace	Negative

**Figure 1:**
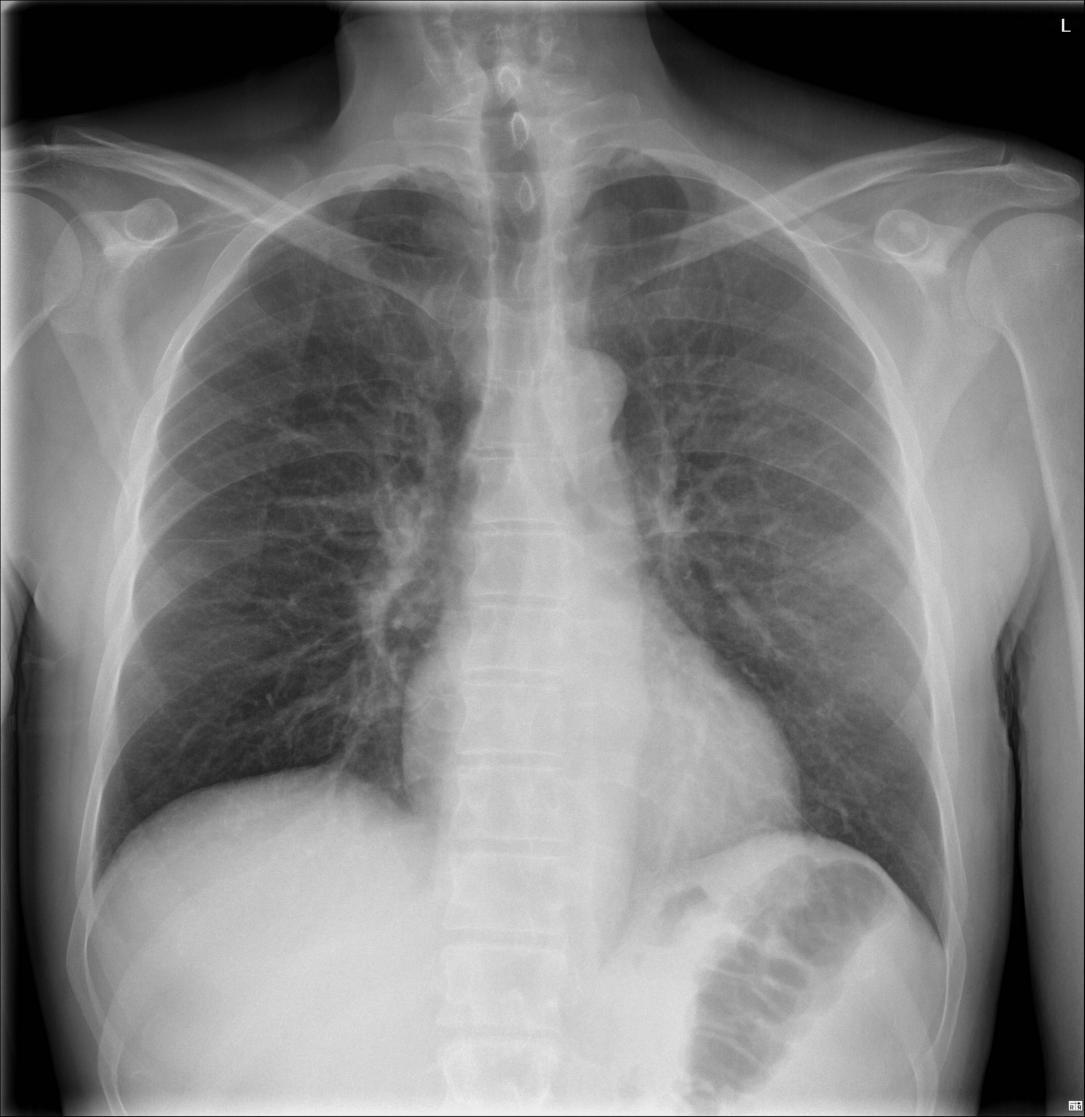
Chest posteroanterior X-ray showing no evident lesions suggestive of right-sided valvular involvement.

**Figure 2:**
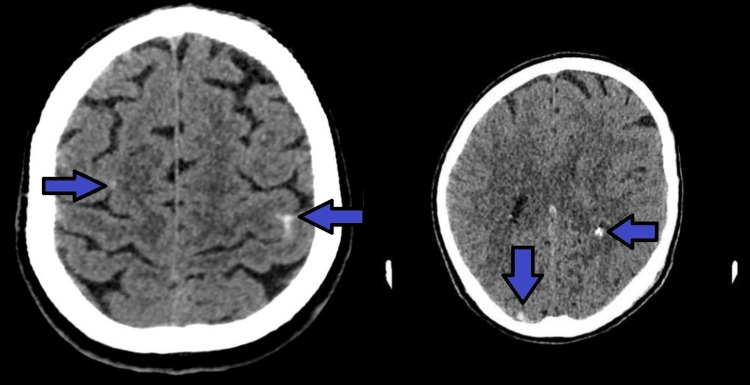
Non-contrast cranial CT scan showing subarachnoid hemorrhage (blue arrows). Image quality limited by patient non-cooperation.

**Figure 3:**
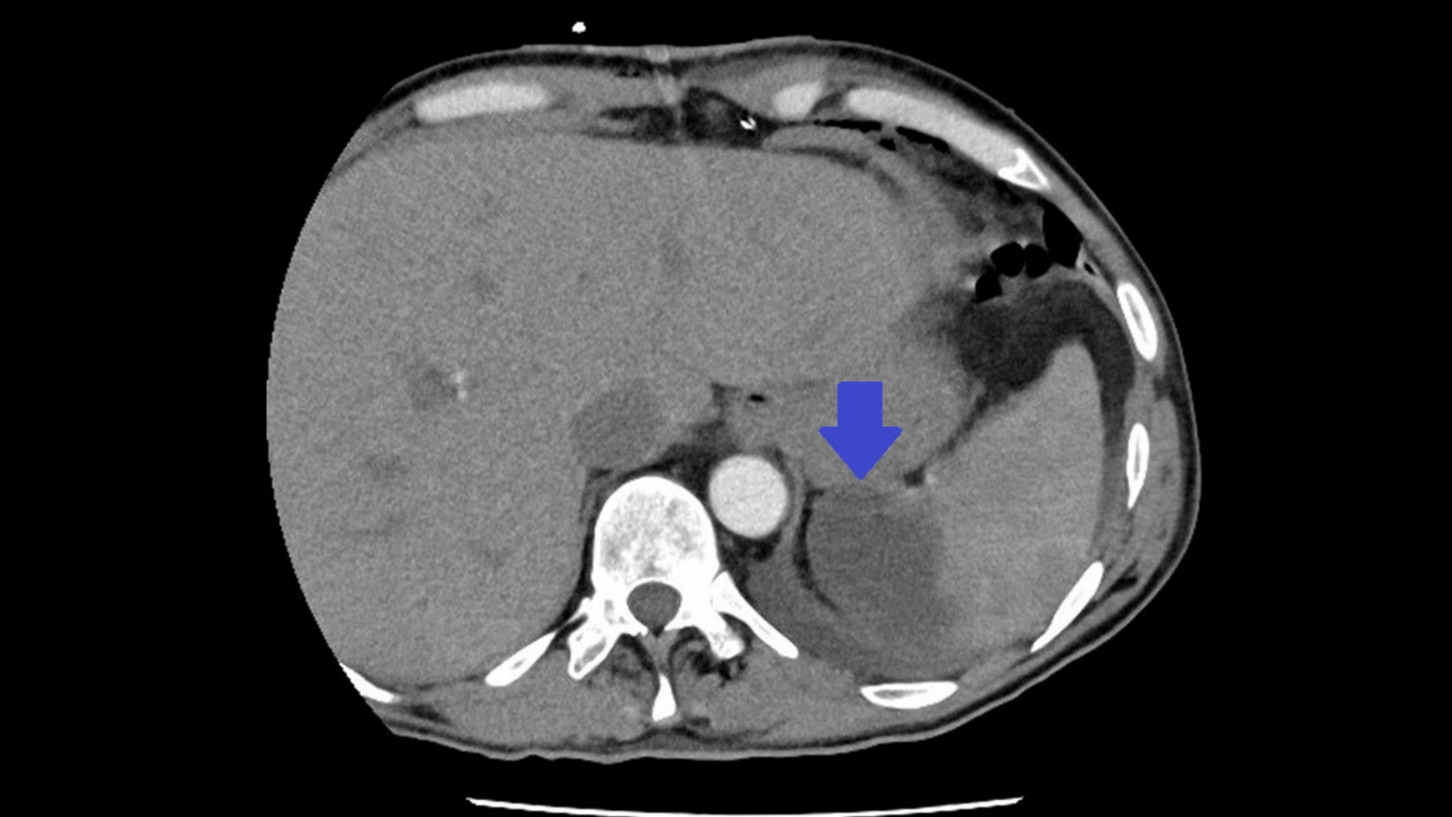
Abdominopelvic CT scan showing splenomegaly and splenic infarction (blue arrow).

**Figure 4:**
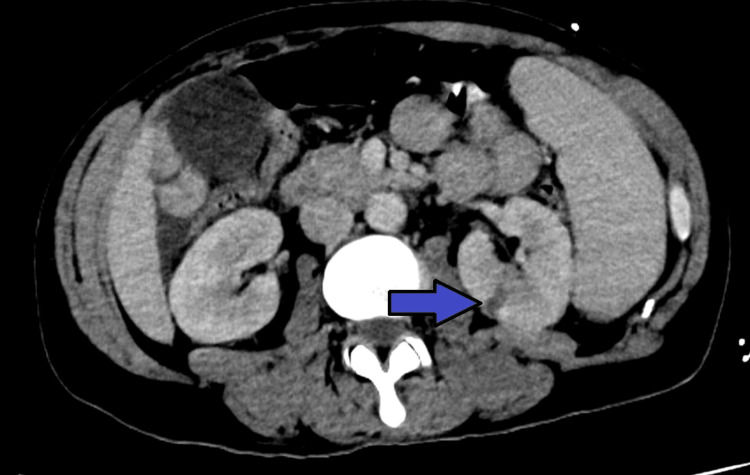
Abdominopelvic CT scan showing a left renal infarction (blue arrow).

Initial chest X-ray did not show clear signs suggestive of pulmonary involvement, supporting isolated left-sided heart involvement in our patient without repercussion on the right heart or lung parenchyma (Figure 1). A non-contrast cranial computed tomography (CT) scan, performed the same day as admission, revealed isolated subarachnoid hemorrhage (SAH) in the bilateral frontal and right occipital sulci (Figure 2). This hemorrhage was presumed to be secondary to septic embolization, as no mycotic aneurysm was identified. Abdominopelvic CT with intravenous contrast demonstrated splenomegaly (16 cm) with a wedge-shaped hypodense area suggestive of splenic infarction, as well as a triangular cortical hypodensity in the left kidney, raising suspicion for renal infarction or focal pyelonephritis(Figures 3, 4). Additionally, small volumes of free fluid were observed in the perihepatic and perisplenic spaces, along with a minimal right-sided pleural effusion.

The patient was admitted to the ICU with a presumptive diagnosis of meningococcemia and meningoencephalitis. The renal and splenic infarctions were initially concerning for thrombotic phenomena; however, they were later attributed to septic embolization related to suspected endocarditis. Therefore, broad-spectrum empiric antimicrobial therapy was initiated, with intravenous ampicillin, meropenem, linezolid, and acyclovir, aiming to cover common bacterial pathogens including *Listeria monocytogenes* and methicillin-resistant *Staphylococcus aureus* (MRSA), as well as potential viral causes such as herpes simplex virus, while awaiting culture results. Adjunctive treatment included dexamethasone, levetiracetam for seizure prophylaxis, and sedation with propofol. Given the marked thrombocytopenia, a platelet transfusion was administered. The patient underwent aggressive fluid resuscitation and was maintained under continuous hemodynamic and neurological monitoring.

On the same day of ICU admission, a lumbar puncture was performed. Cerebrospinal fluid (CSF) analysis revealed pleocytosis with neutrophilic predominance, elevated protein and lactate levels, and normal glucose concentration. Gram stain was negative for microorganisms but demonstrated moderate leukocytosis. Detailed CSF findings are presented in Table 3.

**Table 3:** Cerebrospinal fluid (CSF) analysis performed upon admission to the intensive care unit.

Parameter	Result	Reference Values	Notes
Red blood cells (RBC)	200 cells/μL	0 cells/μL	Elevated
White blood cells (WBC)	70 cells/μL	0 - 5 cells/μL	80% neutrophils
Protein	83 mg/dL	15 - 45 mg/dL	Elevated
Glucose	76 mg/dL	45 - 80 mg/dL	Within the reference range
Lactate	3.6 mmol/L	1.2 - 2.1 mmol/L	Elevated
Gram stain	Negative	—	Moderate leukocytosis present

Preliminary blood cultures obtained shortly after admission grew methicillin-sensitive *Staphylococcus aureus* (MSSA), and urine cultures revealed 25,000 CFU/mL of the same organism. These findings raised suspicion for mitral valve endocarditis as the source of the emboli, which was subsequently confirmed by transthoracic echocardiography (TTE); unfortunately, the echocardiogram images were unavailable and could not be included in this article. The diagnosis was then redirected toward left-sided mitral valve endocarditis with multiorgan involvement due to septic emboli. Antibiotic therapy was adjusted accordingly to include intravenous daptomycin and cloxacillin.

After three days in the ICU receiving targeted antimicrobial therapy and supportive care, the patient showed clinical improvement, hemodynamic stability, and follow-up cranial CT showed near-complete resolution of the subarachnoid hemorrhages, allowing transfer to the internal medicine ward to continue treatment. However, on the second day of hospitalization in the ward, the patient developed recurrent high-grade fever (up to 39°C), raising concern for ongoing systemic infection despite initial improvement.

**Figure 5:**
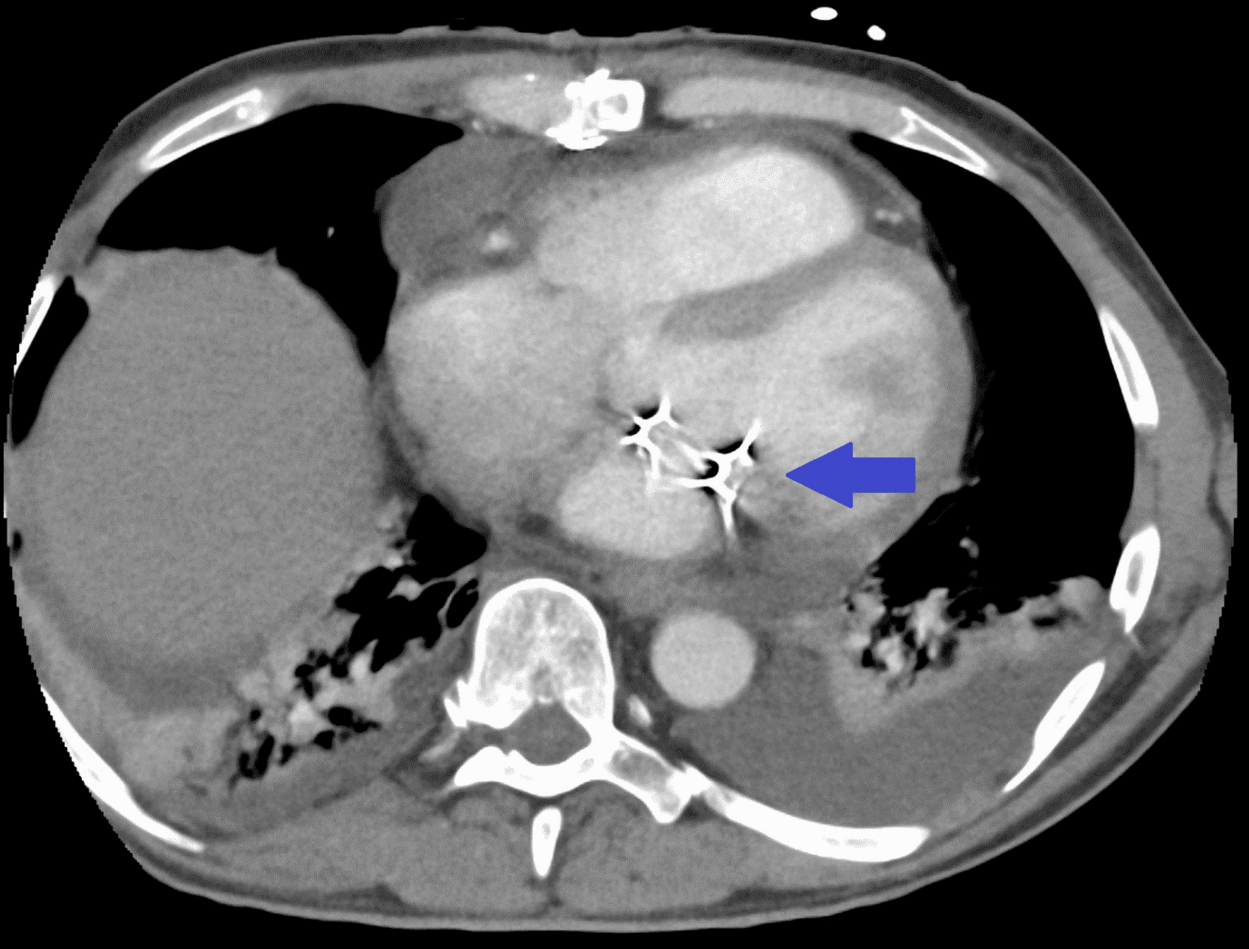
CT scan showing mitral valve replacement with mechanical prosthesis (blue arrow).

Given the confirmed mitral valve involvement, the cardiology team was consulted, and urgent cardiothoracic intervention was indicated in accordance with current recommendations for infective endocarditis with poor response to medical therapy. The patient subsequently underwent surgical mitral valve replacement using a mechanical prosthesis, as repair was not feasible due to the extent of valvular damage (Figure 5).

Notably, the patient’s neurological status improved significantly before cardiothoracic intervention, and no focal neurological deficits persisted, which allowed for surgical planning without delay due to neurologic contraindications.

In the postoperative period, the patient developed transient withdrawal symptoms, which were managed effectively with methadone, supportive measures, and substance use counseling. He completed a six-week course of intravenous antibiotic therapy following surgery while hospitalized. He demonstrated progressive clinical improvement and was discharged in stable condition. At the time of discharge, he had regained his baseline functional status and was referred to a substance use rehabilitation program. Anticoagulation therapy with acenocoumarol was initiated as secondary prophylaxis. Continued follow-up was arranged with the cardiology service to monitor long-term outcomes and valvular function, alongside ongoing implementation of harm reduction strategies such as opioid substitution therapy, counseling, and linkage to community support services to support sustained addiction recovery.

## Discussion

The global prevalence of IE has shown a marked increase over the past decade, accompanied by persistently high mortality rates. The epidemiology of the disease has evolved considerably, driven by demographic shifts such as an aging population, a higher burden of comorbid conditions, and a rising incidence of healthcare-associated infective endocarditis. This increase has been particularly notable in high-risk subpopulations, including individuals with prosthetic valves, immunosuppressed patients, and those who inject drugs. These factors have contributed to a more complex clinical profile and present significant challenges for diagnosis, management, and prevention strategies [8, 9, 10].

IE in IVDU represents an increasingly prevalent clinical challenge, associated with substantial morbidity and healthcare burden. Traditionally, right-sided IE has predominated in this population, largely due to venous injection and the direct exposure of right heart valves to pathogens. However, recent epidemiological studies report a relative increase in left-sided IE cases among IVDUs. Compared to right-sided IE, left-sided IE is associated with more severe clinical outcomes, including higher rates of systemic embolization, heart failure, need for surgical intervention, and long-term disability, as demonstrated in our patient [4, 9, 11].

Unlike right-sided IE, which typically has a more localized and less destructive course, left-sided IE in IVDUs is more aggressive, characterized by larger vegetations, extensive valvular destruction, and a significantly higher risk of systemic embolization. Our patient presented with septic emboli to multiple organs, including renal and splenic infarctions, as well as central nervous system (CNS) involvement manifesting as meningitis and subarachnoid hemorrhage. Importantly, the presence of meningeal syndrome in this patient-with clinical signs such as nuchal rigidity, positive Kernig’s and Brudzinski’s signs, and altered mental status-provided early clues to CNS involvement and highlighted the systemic nature of the embolic process. Affected individuals are generally older, exhibit higher rates of embolic cerebrovascular events, and experience significantly reduced long-term survival. Clinically, left-sided IE often presents with persistent fever, overt signs of congestive heart failure, and systemic embolic complications, frequently leading to elevated in-hospital mortality. Multicenter cohort studies consistently corroborate these findings, demonstrating a stronger association of left-sided IE with congestive heart failure, renal dysfunction, severe valvular insufficiency, and an increased need for urgent valve replacement or surgical intervention [4, 8-16 ].

While IE in IVDUs is traditionally right-sided due to venous return, newer data suggest that left-sided IE is more frequent than previously thought, albeit with a worse prognosis compared to isolated right-sided cases. This particular patient’s presentation was unusual, with CNS infection and severe abdominal pain obscuring the diagnosis [2, 12, 17].

Subsequent imaging confirmed septic emboli in the spleen and kidneys, while clinical and laboratory findings, including positive blood cultures for *Staphylococcus aureus*, supported the diagnosis of meningitis. Although CSF cultures were negative, the presence of meningeal signs and systemic infection strongly suggested septic embolic meningitis due to hematogenous spread. Meningitis is a rare complication of IE, occurring in less than 5% of cases, but its presence warrants suspicion of CNS embolization, especially in patients with altered mental status. These observations align with previous case series and large databases describing the frequency and severity of septic complications, which significantly affect clinical outcomes [7, 9].

Although TTE confirmed the presence of mitral valve vegetations, transesophageal echocardiography (TEE) could have offered superior sensitivity, particularly for detecting smaller lesions or assessing complications such as abscesses. However, due to the clear visualization obtained on TTE and the patient’s clinical instability, a TEE was not pursued. This decision highlights a common clinical challenge where diagnostic optimization must be balanced with patient condition and resource availability.

Intracranial complications such as brain abscesses occur more frequently in acute IE compared to subacute forms. This is largely due to the higher virulence of causative organisms and their capacity for rapid tissue invasion in acute presentations. Moreover, asymptomatic embolic events are frequent; routine neuroimaging may reveal silent infarctions or embolisms in up to 30-40% of IE patients, underscoring the importance of thorough diagnostic evaluation even in the absence of focal neurological deficits [6, 7].

Despite the severity of the valvular infection and embolic phenomena, our patient did not present with clinical heart failure. This may be explained by the early stage of valvular involvement or compensatory cardiac mechanisms preventing overt heart failure symptoms, a phenomenon documented in some case series [9]. The persistent fever and documented septic emboli despite appropriate antibiotic therapy necessitated surgical valve repair in this patient. Postoperatively, the infectious foci were controlled, and clinical improvement ensued, consistent with outcomes reported in the literature [9, 18]. Additionally, cocaine use, reported by the patient, may have contributed to the severity of valvular damage and embolic risk through mechanisms such as vasospasm and endothelial injury, further complicating the clinical course.

Lastly, intravenous drug addiction adds complexity to patient care. Our patient experienced withdrawal symptoms during hospitalization and rehabilitation, emphasizing the need for multidisciplinary follow-up and addiction management to optimize long-term outcomes [18].

This case represented a significant diagnostic and clinical management challenge, requiring careful exclusion of other potential causes. Although urinary tract infection (UTI) with bacteremia was initially considered, it did not account for the imaging findings suggestive of SAH. Disseminated intravascular coagulation (DIC) was excluded, as coagulation parameters remained within normal limits without laboratory evidence to support the diagnosis. Additionally, the patient’s withdrawal syndrome created further uncertainty regarding the origin of his symptoms. Leptospirosis, which could explain the hemorrhagic manifestations and renal involvement, was also ruled out after a negative antigenuria test. Meningococcemia was the leading suspected diagnosis until TTE provided definitive evidence of left-sided mitral valve endocarditis.

One of the limitations of this case is the unavailability of the TTE images. Although the TTE was performed and yielded essential diagnostic information, including the identification of large mitral valve vegetations, we were unable to retrieve the actual images for inclusion in the report, which limits visual documentation of the valvular pathology. Nevertheless, the TTE proved valuable as a non-invasive and readily available imaging modality that contributed to prompt diagnosis and guided initial management.

## Conclusions

IE remains a diagnostic challenge, particularly when it presents with atypical features. This case highlights the need to consider IE in IVDUs, especially in patients with nonspecific infectious symptoms or even when the clinical picture points toward more common conditions in this patient, in whom meningococcemia was initially suspected. Maintaining a high index of suspicion is essential. Prompt recognition, early initiation of broad-spectrum empirical antibiotics, and timely surgical intervention when indicated are critical to improving outcomes. Additionally, comprehensive care must address the underlying substance use disorder, as managing withdrawal and preventing recurrence are integral parts of treatment. Effective management often requires a multidisciplinary approach involving infectious diseases, neurology, cardiology, surgery, and addiction medicine to optimize both short- and long-term prognosis.
